# Survey of Collision Avoidance Systems for Underground Mines: Sensing Protocols

**DOI:** 10.3390/s22197400

**Published:** 2022-09-28

**Authors:** Meilin Qian, Kai Zhao, Binghao Li, Henry Gong, Aruna Seneviratne

**Affiliations:** 1 School of Electrical Engineering and Telecommunications, University of New South Wales, Sydney, NSW 2052, Australia; 2 School of Minerals and Energy Resources Engineering, University of New South Wales, Sydney, NSW 2052, Australia; 3 Roobuck, Suite 6/20 West Street, Brookvale NSW 2100, Australia

**Keywords:** collision avoidance, underground mine, positioning techniques, low-power wide-area communication, medium access control protocol

## Abstract

With the growing number of unintentional interactions occurring in underground mines, Collision Avoidance System (CAS) establishment and maintenance has become an urgent need for mining industries to enhance their risk profile and improve construction safety. Usually, most collision accidents can be divided into three different categories in line with the involved participants and infrastructure condition. The accidents pose a great risk of financial cost to mining companies and even cause casualties. In detail, this paper presents an intensive study survey of positioning techniques, including ranging algorithms, to accommodate the demands of various proximity sensors and improve the capability of situational awareness. Then, we exploit the importance of the communication system, prevalent low-power wide-area technologies and related communication protocols. The effectiveness of communication systems decides and facilitates the success of the final integrated system that can be used to fundamentally address the problem of collision avoidance. For the purpose of collaboration between communication systems and other executive departments, a series of systematic comparisons of pertinent technologies and algorithms is given near the end, followed by a brief discussion on the best choice among these options. In the proposed solution, the overall end-to-end delay can be minimised to a few nanoseconds and the localisation accuracy can achieve centimetre level when operating in the range of 100 m.

## 1. Introduction

The rapid development of mining, especially in underground mining, is driven by the ever-growing demand for mineral resources. There are numerous potential hazards and risks in underground mines. Therefore, a plethora of techniques have been developed and deployed to protect mine workers from risks. Most risks arise as a result of mining workers being in the vicinity of vehicles and vehicles operating in constrained environments: vehicles and personnel (V2P), interactions between vehicles (V2V), and vehicles and mine infrastructure (V2I) [1,2]. Of these, the highest risks are collisions between vehicles and vehicles and personnel [2,3]. In order to minimise these risks, it is necessary to improve situational awareness for mine workers and vehicles by developing automated collision detection systems [4]. The challenge is to provide a Collision Avoidance System (CAS) design that can effectively and accurately take action in confined harsh environments with poor visibility, such as those in underground mines.

**Figure 1:**
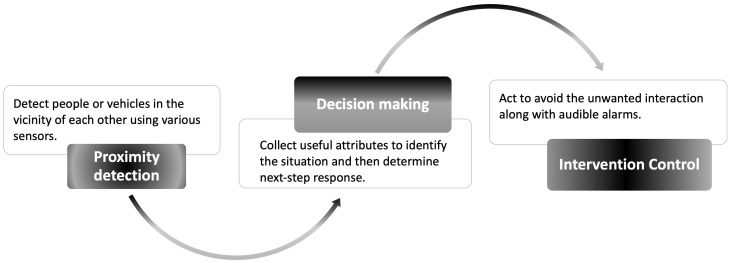
The working flow diagram of a typical CAS.

In general, a CAS consists of three subsystems that need to work together seamlessly, namely subsystems for proximity detection, decision making and intervention control, as shown in Figure 1. The performance of proximity detection relies on sensors to detect objects. The sensors can be visual (e.g., video cameras), ultrasonic, acoustic or electromagnetic (e.g., radar). Actually, proximity detection can be referred to as obstacle recognition as well. If any vehicle/infrastructure in the vicinity can be inspected in advance, and the warning signals can be sent out accordingly, the successful rate of collision avoidance can be improved, and the false alarm problems can be also alleviated [5,6]. Decision-making subsystems consolidate the information obtained from the proximity detection subsystem to determine whether there is an imminent hazard. If there is, it informs the intervention control subsystem to take the necessary actions to avoid the hazard.

The decision-making part is mainly related to the logical judgements and making a final decision based on the instant situation. According to the result of the proximity detection subsystem, it determines whether and how to react. The design of intervention control depends on specific application scenarios and/or the requirements of the local service providers. It is usually composed of audible alarms and vehicle interventions, such as applying the brakes or steering away from the hazard.

It is difficult to carry out a systematic evaluation of the complete situational awareness (collision avoidance) systems that are in use because of the very wide scope of the different subsystems. To perform this, it is necessary to evaluate different subsystems individually. To this end, this paper is mainly focused on the proximity detection subsystem. We perform this by reviewing the ranging algorithms, positioning techniques, low-power and wide-area communication technologies and corresponding sensing protocols. Then, we provide a comprehensive comparison of commercially available techniques and complete systems that can be applied in the mines to prevent collisions. This identifies the advantages and disadvantages of different technologies that are being used and highlights the perspective that requires further development of newer techniques to improve the accuracy, efficiency and usability of these designs, particularly for the environment of underground mines.

The remainder of this paper is organised as follows. Section 2 provides an overview of ranging algorithms and positioning techniques. Then, several prevalent low-power wide-area communication technologies are introduced and compared comprehensively in Section 3. Section 4 presents a survey of available Medium Access Control (MAC) methods suitable for underground mine CAS. It also proposes a series of appropriate evaluation metrics that can be useful for reviewing and comparing existing MAC protocols. In Section 5, we review the critical information for all technologies mentioned in the previous sections and present their advantages and disadvantages. Section 6 provides a pilot system design for collision avoidance and discussion on how the system can be optimised. Finally, Section 7 concludes with a summary and potential directions for future work.

## 2. Ranging Algorithms and Positioning Techniques

Proximity detection can be conducted via different sensing modalities, ranging algorithms or positioning techniques. It is necessary to have seamless coordination between different proximity detection technologies and ranging algorithms that are used in CAS design [7,8]. In general, ranging algorithms are based on the geometrical parameters of the operating environment, such as angles of signal reception and the distance between the sensor and an object. A large number of ranging algorithms have been developed in order to meet various requirements [9,10]. Algorithms that provide high accuracy and can be potentially used for proximity detection in an underground CAS design are introduced in this section, including Time-Of-Flight (TOF), Time-Of-Arrival (TOA), Time-Difference-Of-Arrival (TDOA) and Angle-Of-Arrival (AOA).

### 2.1. Time-Of-Flight (TOF)

TOF uses two-way ranging to measure the distance between a pair of devices. One node acts as the initiator, and the other acts as the responder. The distance can be calculated based on the signal propagation time observed from initiator and responder [11]. The most significant advantage of TOF is not having a requirement for time synchronisation at the initiator and responder, thus minimising complexity of design and implementation [12].

**Figure 2:**
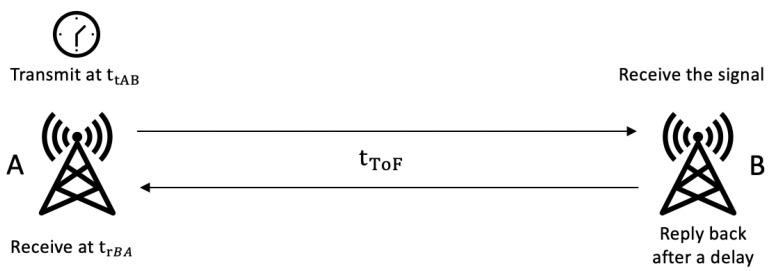
TOF—based distance measurement.

The working principle of TOF ranging is shown in Figure 2. The initiator, node A, transmits a ranging request and records time of transmission ($t_{tAB}$). When the ranging request is received by the responder, node B transmits a reply message back to the initiator. The time taken by the responder to process the ranging request is represented by ($t_{min}$). When the initiator receives the response, it records the time of reception ($t_{rBA}$). Then TOF, based on the clock in the transmitter, is given by the following equation, and the distance between node A and B can be obtained via $t_{TOF}$ times the speed of light,

(1) $$t_{TOF}=\frac{\left(t_{rBA}-t_{tAB}\right)-t_{min}}{2}$$

### 2.2. TOA

TOA uses a transmitter, three noncollinear reference receivers and one-way transmission, where the target is always the transmitter. As the measurements are based on the one-way transmission, TOA relies on a synchronised clock at transmitters and receivers, and the known location of the reference receivers [13,14]. The requirement of synchronisation of the transmitters and receivers is considered to be the major disadvantage of TOA systems. If the transmitter cannot be synchronised, one more unknown (the clock error) parameter needs to be introduced.

**Figure 3:**
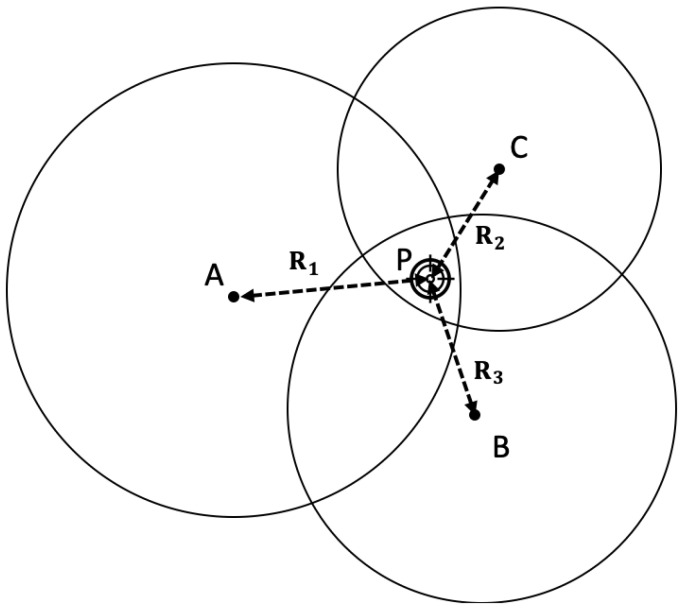
TOA—based distance measurement.

Assume that the coordinates of reference receivers, nodes A, B and C, are $\left(x_{1},y_{1},z_{1}\right)$, $\left(x_{2},y_{2},z_{2}\right)$ and $\left(x_{3},y_{3},z_{3}\right)$. Then, set the coordinate of the target object and the transmitter P as (*x*,*y*,*z*), as shown in Figure 3. At first, a ranging request is sent by P, with a record of the time of transmission. Receivers A, B and C then calculate the distance from themselves to P $(R_{1},R_{2}$ and $R_{3})$ using time difference between the transmitting time and the receiving time. The position of the transmitter P (x,y,z) is calculated as follows:

(2) $$R_{1}=\sqrt{{\left(x-x_{1}\right)}^{2}+{\left(x-x_{2}\right)}^{2}+{\left(x-x_{3}\right)}^{2}},$$

(3) $$R_{2}=\sqrt{{\left(y-y_{1}\right)}^{2}+{\left(y-y_{2}\right)}^{2}+{\left(y-y_{3}\right)}^{2}},$$

(4) $$R_{3}=\sqrt{{\left(z-z_{1}\right)}^{2}+{\left(z-z_{2}\right)}^{2}+{\left(z-z_{3}\right)}^{2}}$$

### 2.3. TDOA

The operation of TDOA is similar to TOA, except that TDOA uses the cross-correlation of signals at two reference receivers to measure the distance from target as described below. Alternatively, TDOA can obtain the location of target P through the difference of distances at each reference receiver node [15,16].

**Figure 4:**
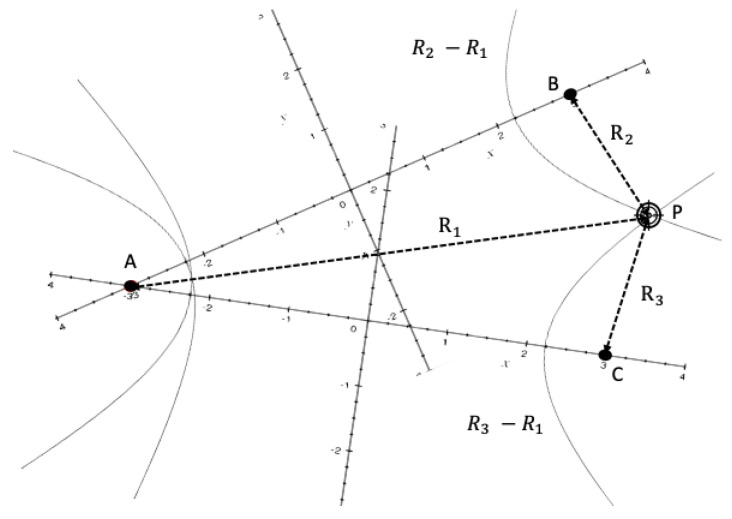
TDOA—based distance measurement.

TDOA only requires time synchronisation of the reference receivers [17,18]. The target P is at the intersection of two hyperbolic curves that maintain an identical range difference between different preset receivers and the same target, i.e., *R*_2_ − *R*_1_ and *R*_3_ − *R*_1_ are equal and constant, as shown in Figure 4. The first hyperbola is associated with nodes A, B and target P, and the second hyperbola is associated with nodes A, C and target P. The target P sends out the first ranging signal ($S_{t}$), which is known by node A and B. After a period of transmission, there must be certain changes in this ranging signal when reaching node A and B. The actual signals received by A and B can be denoted by $S_{rA}$ and $S_{rB}$, respectively. By determining the maximum of correlation between $S_{rA}$ and $S_{t}$ and $S_{rB}$ and $S_{t}$, the time taken for the signal to travel from P to A, and P to B, can be determined. Similarly, the time taken for the signal to travel from P to A and to C can also be determined. Then, use the cross-correlation equation to estimate TDOA:

(5) $${\hat{R}}_{21}\left(\tau \right)=\frac{1}{T}\int_{0}^{T}S_{t}\left(t\right)\cdot S_{rA}\left(t-\tau \right)dt$$

### 2.4. AOA

AOA uses the arrival angle of the signal transmitted by target P at two predefined reference nodes A and B to determine the location of P. The angle measurements rely on antenna arrays. The target P must lie at the intersection point of two direction lines formed by the angle determined from each reference node. This method takes only two sensor nodes compared with TOA and TDOA, which require at least three reference nodes (for 2D positioning). However, the accuracy of the AOA mechanism used in Bluetooth devices is not as high as TOF, TOA and TDOA.

**Figure 5:**
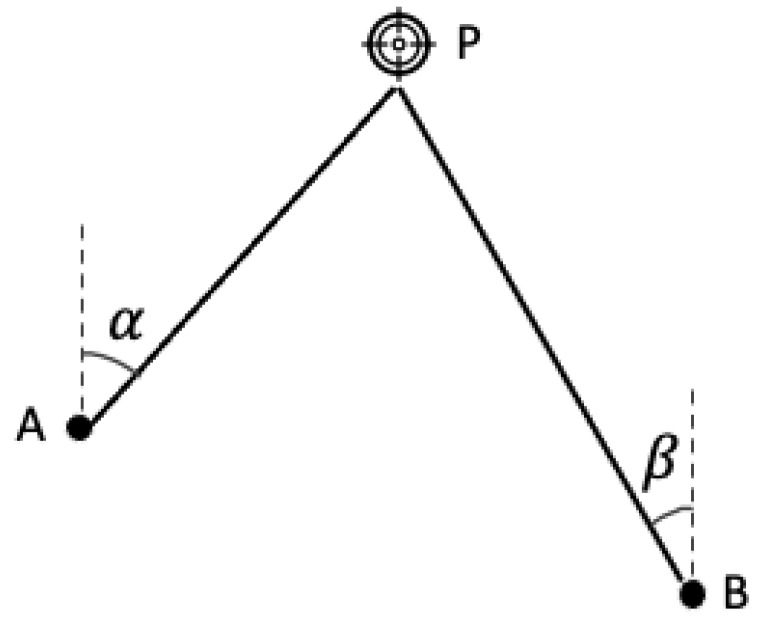
AOA—based distance measurement.

The operation of AOA is shown in Figure 5. Again, the target P sends out a signal. Nodes A and B determine the angle of arrival (incidence) for the signal (i.e., α and β). Assume the locations of two reference nodes A and B can be represented with ($x_{1},y_{1}$) and ($x_{2},y_{2}$). Then, based on the following basic geometric knowledge, it is possible to determine the location of P (x,y) through a series of iterative matrix operations.

(6) $$tan\theta_{1}=\frac{y-y_{1}}{x-x_{1}}$$

(7) $$tan\theta_{2}=\frac{y-y_{2}}{x-x_{2}}$$

## 3. Communication Requirements

A proximity detection system in an underground mine always needs to rely on multiple sensors mounted on vehicles and mine workers communicating with each other. This imposes some strict requirements on the communications between these devices, such as not having collisions and having minimum latency. The communication system further needs to be capable of operating with minimum power supply. The communication devices in the vicinity can exchange information with each other prior to the transmission of the proximity sensors’ data.

**Figure 6:**
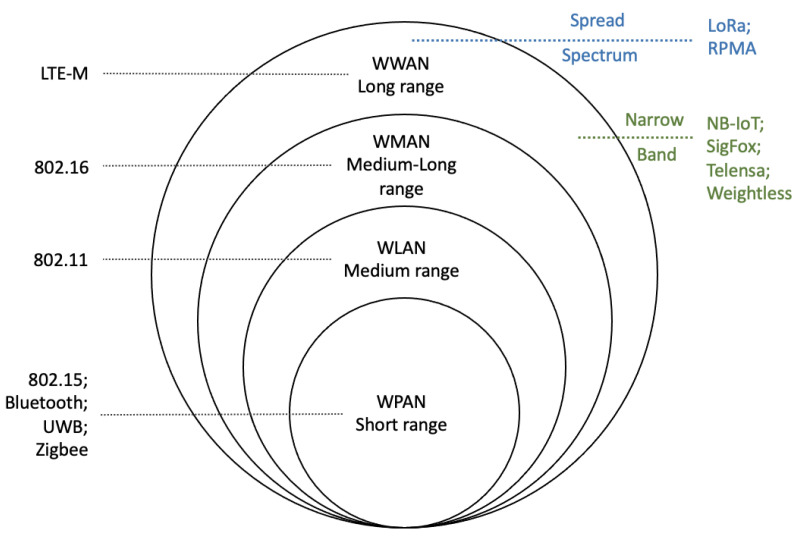
Distribution of LPWA technologies in general wireless networks.

Low-power wide-area (LPWA) communication technologies have evolved rapidly in recent years with the development of the Internet of Things [19,20]. The benefits of the LPWA technique, as the name suggests, are low power consumption and stable and qualified connection capability over long range (i.e., a few kilometres) at low cost [21,22,23], and it can operate in different frequency ranges. Based on their different coverages, the relationship between LPWA technologies and general wireless networks is shown in Figure 6.

A Wireless Personal Area Network (WPAN) provides short-range communication (i.e., approx. 10–100 m). The most widely deployed WPANs are Zigbee and Bluetooth/Bluetooth Low Energy (BLE) [24,25], which operate at the 2.4 GHz unlicensed frequency band in most cases. These two wireless communication techniques both have the benefit of low power consumption, whereas the retransmission rate has no impact on the power consumption of Zigbee. Bluetooth/BLE works in an opposite way. Longer transmission distance results in more packet errors and higher retransmission rates, which increases the total energy consumption. In most wireless IoT applications, the typical power consumption of Zigbee and BLE devices are between 10 to 100 mW, which is 10 to 100 times less than traditional Bluetooth devices.

The Wireless Local Area Network (WLAN) supports medium signal coverage (i.e., approx. 100–1000 m). The most widely used WLAN technologies are based on the IEEE802.11 standard and operate at 2.4 GHz or 5 GHz. The WLANs enable the connection at significantly different levels of energy consumption, depending on the actual network element selection. In general, there are three different network elements that consume power, including Access Points (APs), station/controller and distribution system (consisting of a large amount of switches). Each AP typically draws up to 10 Watts travelling through one hop only.

The Wireless Wide Area Network (WWAN) is suitable for long-range communications, up to 100 km [26]. The most widely used WWANs include Long-Term Evolution-Machine Type Communication(LTE-MTC), Narrow-band Internet of Things (NB-IoT) and Long range (LoRa). LTE-MTC and NB-IoT rely on access to Long-Term Evolution (LTE) networks that operate at 1.4 MHz and 180 kHz, respectively [27]. More recently, propriety communications systems such as Sigfox, Telensa and Weightless have also become popular [27].

The WWAN technologies, with the possible exception of LoRa, are not suitable for underground mines due to their reliance on communication infrastructure above ground such as cell towers. Whilst it is possible to obtain portable base stations *KUHA*, they are expensive and inflexible due to their reliance on licensed frequency bands [28]. As a result, they are not used for underground applications.

**Figure 7:**
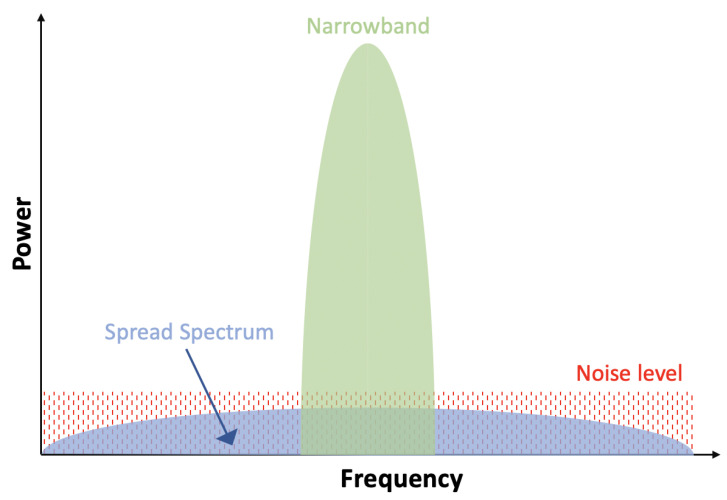
Narrowband vs Spread Spectrum.

There are two dominant communication techniques in the LPWA communication field, namely Narrow Band (NB) and Spread Spectrum (SS), as shown in Figure 7. As the upper limit of output power is usually stipulated by government regulations, the communication range is dictated by data rate [29]. In other words, higher data rates contribute to a decreased sensitivity at the receiver but reduce the probability of interference throughout the transmission [30]. Thus, there is a trade-off between range and transmission rate.

### 3.1. Narrowband

Narrowband technology utilises narrow radio frequency channels to enable high receiver sensitivity and extremely long-range reach at a relatively low data rate [31]. Typically, a narrowband system separates the transmission channel for uplink and downlink communications using different frequency bands. The narrowband technique usually allows frequency-division-based multiple accessibility via channelised frequency spectrum. The narrowband communication system has the demerit of low and fixed data rates with restricted applicability [32], whereas its merit is easy implementation with low deployment density [33]. Nowadays, there are several specialised narrowband technologies such as Sigfox, Telensa, Weightless, NB-IoT, etc.

#### 3.1.1. Sigfox

Sigfox is a reliable ultra-narrowband solution using unlicensed sub-1 GHz Industrial, Scientific and Medical (ISM) band. Sigfox offers wide area connectivity and is suitable for small data transmission [34]. Usually, the user experience of services provided by Sigfox primarily depends on the gateway function controlled by local network providers who are responsible for supplying regional supporting networks. The downlink channel is reported to not be as effective as the uplink transmission channel [35].

#### 3.1.2. Telensa

Similar to Sigfox, Telensa is another narrowband technology for low-power, low-rate and long-range communications using the unlicensed sub-1 GHz ISM frequency band. In contrast to Sigfox, Telensa fully supports bidirectional communication. This enables Telensa to be used in control centres as well as systematic monitoring [36]. To date, a number of wireless nodes have been deployed in smart city infrastructure across more than 30 countries [37].

#### 3.1.3. Weightless

Weightless provides several distinctive characteristics which are not available in Sigfox and LoRa [38], and it was developed by Weightless Special Interest Group as an open public standard for low-power, low-rate and long-range wireless networks. In total, there are three different standards, namely Weightless-N, Weightless-P and Weightless-W [39]. Weightless-N aimed at providing one-way communication at ultra-low cost. Weightless-P strove for bidirectional communications. It provides higher performance and communication reliability [40]. Weightless-W is specifically devoted to whitespace when the frequency spectrum occupied by TV becomes available. Due to the advantages of low-latency and low-energy consumption, Weightless-N is the most suitable option for the CAS design in underground mines [38].

#### 3.1.4. Narrowband Internet of Things (NB-IoT)

NB-IoT is introduced by the 3rd Generation Partnership Project (3GPP), which is based on cellular networks and aimed to improve IoT connectivity. It can be deployed on the Global System for Mobile Communications (GSM) network, Universal Mobile Telecommunications System (UMTS) network or Long-Term Evolution (LTE) network, thereby no extra cost or implementation for upgrade is required [31]. The main features of NB-IoT are wide coverage, multiple accessibility, low data rate, low cost and extended battery lifetime [41]. In contrast to other ultra-narrowband techniques, NB-IoT occupies the licensed frequency band, and as a result, leads to large costs if the spectrum has to be purchased.

### 3.2. Spread Spectrum

Spread spectrum technology has been widely used in the military, industrial, and scientific area for a long time [42]. A typical spread-spectrum-based communication transmits data by spreading it with low power over a bandwidth that is much larger than the frequency band necessary to transmit the raw data signal. There is only one radio frequency channel for both uplink and downlink communications to share [37]. The biggest advantage of spread spectrum is the ability to transmit signals with a low spectral power density using varying carrier frequencies at the same amount of transmit power [37]. Overall, two of the most popular LPWA communication systems involved with spread spectrum technology are Long Range (LoRa) and Random Phase Multiple Access (RPMA).

#### 3.2.1. Long Range—LoRa

It was developed by the LoRa Alliance [39]. LoRa is based on spread-spectrum modulation techniques derived from chirp spread-spectrum technology. The technology was acquired by Semtech, the founding member of the LoRa Alliance and patented. This LoRa is a proprietary LPWA network modulation technique [43]. Typically, LoRa occupies sub-1 GHz ISM bands and can be either private by paying local network providers for any special request or public by applying a unique node identification through the LoRa Alliance.

#### 3.2.2. RPMA

RPMA is also a spread-spectrum technique developed by Ingenu. It operates on an unlicensed ISM band and supports optional encryption function for secure communication. RPMA is in use across more than 20 countries and is capable of inter-connecting most commercially available IoT devices seamlessly [44]. Using RPMA at 2.4 GHz ISM frequency band can maximise the transmission range at the stipulated transmit power but suffers from the co-existing problem if employed with commercialised Wi-Fi devices [45]. Specially, RPMA technology has an advantage of data rate adaptability [39] that can vary with the spreading factor based on the signal intensity in downlink transmissions and uplink channel condition. For example, the channel signal information retrieved from the received uplink message can be employed to determine the downlink data rate [46].

## 4. Medium Access Control Methods and Communication Protocols

### 4.1. Desired MAC Protocol Design for CAS

In terms of a successful CAS design, proximity detection based on different detection capabilities is required. Correspondingly, a suitable communication system is also important, which is used to arrange multiple sensor nodes to perform proximity detection in order. A complete communication system needs to have advanced communication technology that is suitable for low-power and long-range networks (i.e., LPWAN). The system also relies on an appropriate MAC protocol to share the common communication channel without packet collisions and exchange information with each other with minimum latency.

The frequency channels occupied by the signals used in the proximity detection systems and the communication systems need to be separated to avoid mutual interference. This can be achieved using three techniques, namely Frequency-Division Multiple Access (FDMA), Code-Division Multiple Access (CDMA), or Time-Division Multiple Access (TDMA). In the CAS design for underground mines, the channel access opportunity for each node has to be scheduled in advance in order to avoid packet collisions. Therefore, FDMA and CDMA are not suitable for underground applications in most cases due to the limited bandwidth that can be provided in the underground mining environment [47,48]. Additionally, CDMA relies on complex modulation, which increases the difficulty of implementation [49,50]. As a result, TDMA, using fixed assignment of time slots, offers the highest probability to be implemented in underground applications [51,52].

In order to create and maintain a contention-free communication channel, TDMA provides a way of time slot distribution for all devices in the neighbourhood. In order to have an organised time schedule without any packet collision, it is necessary to consider the following features and requirements for a desired medium access control method. As shown in Table 1, the importance of each characteristic and some typical examples of existing MAC protocols that are unfolded later are both included.

#### 4.1.1. Distributed Networks

In terms of a ranging-based proximity detection systems, multiple devices need to interact with each other and use peer-to-peer connection. Particularly, when using the TOF strategy to perform two-way ranging, a star network topology would be preferred if vehicles/personnel are close to each other. In such a setting, the ranging request will be initiated by a master node and received by the rest of the nodes individually.

#### 4.1.2. Contention-Free Communication

The communication between the devices needs to be reliable and efficient regardless of the number of the involved vehicles/personnel. In addition, each round of ranging process has to be completed without any interruption. This requires that the transmission of ranging signals needs to be collision free.

#### 4.1.3. Scheduling Protocols

In an underground mine with moving vehicles and mine workers, a flexible scheduling mechanism that assigns each time slot to the appropriate device is required. Furthermore, the system needs to accommodate numerous application scenarios including both long straight tunnels and confined corners, regardless of the size of the coverage area. In addition, several critical parameters need to be taken into consideration, such as the maximum time delay, network capacity and energy efficiency.

#### 4.1.4. Latency

One of the most important features of a CAS system is latency. Latency depends on the time delay used to contend for channel access and the time taken for message delivery.

#### 4.1.5. Energy Consumption

In order to have a successful CAS design for underground mines, a multitude of proximity detection sensors and communication devices are necessarily deployed over a vast area. Due to the hostile environments and limited power supply in the underground mines, energy consumption is normally the first priority to be considered in order to keep the network composed of various devices working over a relatively long period with no need for regular maintenance or battery exchange. Although, if all devices are mounted on either vehicles/machinery or personnel rather than fixed infrastructure, the problem of limited power supply can be resolved easily without extra effort. In that case, energy consumption would be viewed as a second important factor only to over latency. In fact, energy efficiency as a critical evaluation metric among abundant available MAC protocols also has an interactive relationship with end-to-end delay, packet delivery reliability and channel maximum throughput. Based on a thorough understanding of power dissipation in different MAC protocols, there are three causes, namely overhearing, overhead and duty-cycling, to be further discussed below.

#### Overhearing

During the packet delivery phase, a node may hear signals frequently from the sender in the neighbourhood, whereas it is not the destination of the forwarded data packets. Even though idle listening without further process consumes less power than the intended node, the energy waste cannot be ignored since the sleep state of the same node saves much more energy than the idle listening mode.

#### Overhead

A sequence of control packets such as RTS and CTS may contribute to large overheads that consume a considerable portion of energy, which decreases energy efficiency significantly. Alternatively, various preambles, including short and long preambles, increase power consumption to a great extent. Particularly, long preambles do not convey useful information but occupy the limited resource of applications.

#### Duty-Cycling

In essence, the radio of sleep period and wake-up period determines the duty-cycle of MAC protocols. A sensible duty-cycling is capable of maximum energy efficiency and minimum end-to-end delay. A short duration of sleep period leads to intense power consumption, whereas a too long sleep scheduling can potentially cause a large time delay to wait for the response from the next hop that is in sleep period.

#### 4.1.6. Scalability

In terms of the application scenario in the underground mines, there might not be many vehicles and personnel in the proximity. However, in other complicated applications, such as an intelligent mobile app used to keep physical distance during the COVID-19 situation, there probably is a need to consider the network scalability when designing a suitable MAC protocol. In order to make the designed MAC protocol with enhanced adaptability and flexibility to accommodate different kinds of applications, the system scalability should be paid attention to appropriately.

#### 4.1.7. Traffic Adaptability and Throughput

In terms of an effectual communication protocol, some other evaluation metrics are worth mentioning, though they are not critical factors to be considered for CAS design in underground mines. Traffic adaptability provides a potential way for a flexible time scheduling mechanism to further improve energy conservation and reduce time delay, respectively [53,54]. Additionally, channel throughput cannot be ignored as well. Based on limited frequency resources and a plethora of devices deployed in some applications, it is necessary to have an exceptional performance in system throughput via the employment of variable scheduling mechanism and minimised possibility of packet collisions [55].

#### 4.1.8. Handling Mobility

The mobility awareness provided by MAC protocols specially caters to dynamic sensor networks. Adjusting resource allocation to ensure the fairness of distribution among all the deployed devices based on real-time mobile nodes is essentially important in CAS design, since most vehicles and personnel in the underground mine are usually in the non-stationary status. An accurate estimation of actual traffic flow and the number of involved nodes can provide the communication network with maximum channel efficiency and system capacity.

#### 4.1.9. Wake-Up Radio Enabled

The communication system is designed to focus on time frame arrangement so that the proximity detection process can be conducted during corresponding time slots. Using external devices to reach different targets is also a useful algorithm when designing a suitable MAC protocol for communication systems. In other words, the deployment of extra wake-up devices on the side to assist the main communication device to have high sensitivity in time scheduling catering for dynamic wireless nodes.

### 4.2. Review of Existing Wireless Standards and MAC Protocols

In underground mine CASs, a large amount of wireless sensors are deployed, and the communications with these sensors over the shared communication channel need to be separated from each other. In other words, it is necessary to have a suitable medium access control (MAC) scheme, which will enable all involved distributed devices to be effectively managed.

#### 4.2.1. Related Wireless Standards

The IEEE 802.11ah and IEEE 802.15.4 standards are capable of supporting efficient wireless long-range communication at low power consumption. The biggest difference between 802.11ah and 802.15.4 is the number of wireless devices that they can support [56].

##### IEEE 802.11ah

The IEEE 802.11 Working Group for wireless local area networks developed the first wireless standard, and that standard has evolved to support high-speed, wide coverage [57] and transmission technologies, especially Multiple Input Multiple Output (MIMO).

The latest standard, IEEE 802.11ah, supports low-power sensor network communication. It has also been adapted for long-range communication among a large number of end devices [56] using Relay APs. IEEE 802.11ah operates on the sub-1 GHz unlicensed frequency band to enable an extended operation range, and specially designed data structures are used to minimise the power consumption [34].

##### IEEE 802.15.4

WPANs are usually used to convey information privately over short distances among an intimate group of participant devices. They are dedicated to providing an effectual wireless communication approach within the personal operating space; a circular operation with a radius of 10 m centred on the primary device [58].

Typically, there are two types of WPANs, namely high-rate WPAN and low-rate WPAN. The IEEE 802.15.4 standard is particularly aimed for low-rate WPAN, and it provides corresponding lower-power physical and MAC-layer provisions. This standard contributes to the development of industry standards such as ZigBee and Bluetooth, which are mainly used for low data rate transmission at low power consumption. IEEE 802.15.4 uses the unlicensed 2.4 GHz frequency band and Direct-Sequence Spread Spectrum (DSSS) modulation scheme. DSSS employs a chipping code to “spread” the transmission over a wider frequency band than it would normally occupy [59]. In addition, the super-frame structure and sleep–wake strategy might be useful for this standard to improve energy efficiency.

#### 4.2.2. Typical MAC Protocols

In order to have a high-performance CAS design for underground mines, a detailed table that reviews various MAC protocols is provided in Section 7. Moreover, a brief discussion of a few typical MAC protocols is given in different groups based on their different characteristics.

#### Latency

- **SR-MAC**

SR-MAC [60] is a synchronous MAC protocol with SYNC, DATA and SLEEP phases. Most of the existing medium access control (MAC) protocols for sensor systems are mainly optimised for the situation under which a device only generates one packet. As a result, when multiple packets are generated by a device, the performance of these MAC protocols degrades. SR-MAC overcomes this by using a three-phase operation: SYNC, DATA and SLEEP phases. It introduces a new scheduling mechanism that reserves few time slots during the SLEEP period to enable devices to transmit multiple packets, which allows the scheduling of multiple packets generated by a device to be transmitted in one operational cycle without collision.

SR-MAC uses a slot-reservation mechanism during the SLEEP phase of an operation cycle to schedule wake-up nodes to communicate. In the DATA phase, a device that wants to transmit data packets contends for the channel access using a CSMA/CA protocol. SR-MAC replaces RTS/CTS with a special control frame, called the slot-reserved frame (SRF). In the SLEEP period, according to the slot in which the node transmits the SRF, the neighbouring devices wake up to communicate with each other in the corresponding slot.

In the DATA phase, each pair of frame-based slots, and further-divided subslots in the third phase, are linked to a corresponding time slot in the second phase. The design of the Slot-Reserved Frame (SRF) instead of the Request-to-Send (RTS) and Clear-to-Send (CTS) frames, particularly for a receiver, enables not only the follow-up reservation deployment for itself but also a new reservation request for the next hop in the forwarding path. The enhanced scheduling mechanism provides multi-packet transmission over multiple nodes using cross-layer routing information to further decrease packet delivery delay. Additionally, in order to maintain energy conservation, the information of SRF is capable of informing only the involved nodes to wake up during the third phase and keep the rest of the irrelevant nodes with the least power consumption. However, the maximum number of packets to be transmitted in one operational cycle is limited and determined by the duration of frames and the number of subslots segmented within each frame.

- **SW-MAC**

Different from SR-MAC, SW-MAC [61] is an asynchronous low-latency MAC protocol with adaptive duty-cycle. The duration of wake-up and sleep periods is determined by real-time data rates or traffic congestion. In order to shorten the end-to-end time delay across multi-hop transmission, the employment of scout packets instead of long preambles facilitates the wake-up and sleep scheduling for corresponding nodes to be performed promptly. The scout-based scheme actually behaves as a triggering signal, which is similar to various preambles, and it solves the problem of large overheads by dividing them into small pieces, then encapsulating them into a series of wake-up packets. Furthermore, the Additive Increase/Multiplicative Decrease (AIMD) mechanism is utilised to adjust the duration of sleep state. It is extremely suitable for wireless network traffic with large variance by minimising the long waiting time caused by the next hop, since it is still in sleep period. In addition, SW-MAC has no stringent requirement for time synchronisation among a large number of nodes via alleviating the serious issue of heavy overheads occurring in the multi-hop forwarding process. The energy consumption is reduced impressively. The flexible duty cycle also provides energy conservation by adjusting the sleep-wake scheduling mechanism based on actual network traffic conditions. An important limitation of SW-MAC is the assumption of only one source node to generate packets for detected events that have been made upfront. In underground mines, there must be more than one vehicle with devices mounted inside working as the source nodes in actual application scenarios.

- **DW-MAC**

A synchronous duty-cycle MAC protocol providing low-latency capability based on considerable energy efficiency was proposed, called Demand Wakeup MAC (DW-MAC) [62]. It introduces a novel sleep–wake scheduling mechanism that allows nodes participating in the communication to convert between sleep and wake-up period on demand. DW-MAC has an exceptional performance when applied in congested networks with heavy traffic loads or large data rates due to its adjustable duty-cycle scheme. It makes use of the scheduling frame (SCH) to replace RTS/CTS and schedule nodes to wake up or fall asleep within each operational cycle. SCH works collaboratively with the designed mapping function to reserve corresponding time slots in the subsequent sleep period over the same duty cycle. The optimisation method using SCH for multi-hop packet delivery with reduced latency is typically dedicated for broadcast operation mode, although DW-MAC can be adjusted to be compatible with the unicast mode as well. Overall, the time delay reduction method in this work mainly relies on the flexible sleep-wake scheduling mechanism, though DW-MAC enables significant energy conservation in addition to the relatively short time delay. The mapping function provided by SCH resolves the problem of hidden terminals so that conflict-free communication with reduced overheads is possible. However, the drawback of DW-MAC is obvious when compared with SR-MAC: there is only one packet that can be transmitted over each operational cycle, and multiple packet delivery is frequent to see in terms of event detection using wireless sensor devices. It has an essential impact on the final end-to-end delay and system capacity.

- **LDC-MAC**

The work of [63] presents a low-latency MAC protocol that is suitable for dual-channel communication networks. The time synchronisation over two independent transmission channels leads to extra energy consumption, which is a serious problem to be resolved. Meanwhile, the overall time delay could be potentially increased as well. Based on these facts, the design of the base station with no constraints in power supply was introduced in LDC-MAC [63] to schedule all the other nodes to deliver packets or keep idle listening from the perspective of global control. The duty-cycle of LDC-MAC that determines the duration of sleep and wake-up period can be adjusted for each sensor node according to the predefined packet forwarding path. Consequently, the latency is decreased because of the reduced waiting time caused by the sleeping state of the next receiver. The sleep–wake scheduling mechanism also has an impressive effect on energy conservation. Unfortunately, dual-channel communication systems are usually not available in underground mine devices. Most cost-effective communication devices that are suitable for underground mine environments have constrained resources either in frequency selection or channel bandwidth.

#### Energy Consumption Due to Overhearing

- **BBAD Mechanism**

In the wireless personal area networks with wake-up radio devices enabled, the validation process for the devices addresses was enhanced in [64] in order to resolve the overhearing issue and further improve energy conservation. The introduced method is based on a preset decoding scheme which process each address bit by bit with minimum possible error rate. The validation result based on the Bit-by-bit Address Decoding (BBAD) mechanism is reliable to avoid confusion, even when faced with abundant devices, but aims for only one node. The BBAD process is conducted within the intended receiver only, and it turns to sleep period automatically whenever one error bit occurs to save energy consumption.

- **RANO Mechanism**

An effective approach was proposed in [65] to inform each tag device of its time schedule, including active periods and inactive periods to improve energy efficiency via avoiding the overhearing problem. The Reservation Aloha for No Overhearing (RANO) mechanism can be implemented on each access point regardless of the network architecture (i.e., with or without a central node). The overhearing occurring on unintentional nodes can be resolved through a reservation and error recovery mechanism; the information for reservation and recovery is designed to be displayed by a particular byte representation to perform a comparison check. The RANO scheme works with a preset assumption that all involved nodes have been time synchronised accurately, which can be counted as a strict requirement. This protocol is designed for active Radio-Frequency Identification (RFID) tag devices and aims for energy waste caused by the overhearing problem. It implies an obvious advantage of significantly enhanced energy efficiency.

#### Energy Consumption Due to Overhead

- **LO-MAC**

The MAC protocol proposed in [66] is designed for wireless sensor networks with low data rates, and it provides a low-latency, low-overhead and energy-efficient medium access control method. The Pioneer (PION) packet is employed to replace the common control packet of RTS or CTS in order to mitigate overheads. The goal of PION packets is to initialise the connection among different nodes, and it actually includes cross-layer information that is useful for multi-hop transmission. The PION packet plays an important role in scheduling nodes to sleep or wake up separately using the nature of broadcasting to send control packets with different meanings. The LO-MAC [66] also makes use of duty-cycling and the optimised multi-hop routing algorithm to reduce latency. Additionally, an adaptive sleep–wake scheduling mechanism was incorporated with the Carrier Sense Multiple Access (CSMA)-based contention scheme to further improve energy efficiency and channel utility.

- **LoBigMAC**

In [67], a MAC protocol using the TDMA technique for data transmission and CSMA mechanism to contend for channel access was proposed. It provides a low-delay, reliable and power-efficient medium access control method using a receiver-initiated scheme to extend the network battery lifetime. A unique feature of this MAC protocol is its network architecture, which is a tree model. In order to construct a successful tree-shape network, time synchronisation has to be performed first. Then, a preset big shareable slot can be segmented and assigned to nodes on different levels in the tree model. Since only the nodes at the same level contend with each other to obtain channel allocation to send packets, differentiating nodes into various levels plays an essential role in reducing the number of control packets. Meanwhile, the structure of each divided big shareable slot is designed to minimise the packet collision rate. Consequently, the overhead effect is diminished, and power efficiency can be enhanced significantly.

- **LCO-MAC**

The LCO-MAC [68] is another typical protocol that focused on the overhead problem. It provides a reasonable solution for energy conservation based on the trade-off issues caused by the duty-cycling mechanism in most MAC protocols. To decrease the number of control packets, each packet is generated for multiple purposes that are different for uplink and downlink transmission. During the initialisation phase, the same control packet behaves as an RTS packet to be sent to the receiver node, whereas it acts as a CTS packet when transmitted to the sender node from the receiver end. Within the procedure of data transmission, the control packet with the same contents is capable of representing acknowledgements as well. However, it is only enabled in uplink transmission, but remains the original meaning during the downlink communication channel. In addition, it enables multi-hop transmission within each operational cycle to reduce packet delivery latency.

#### Energy Consumption Due to Duty-Cycling

- **BN-MAC**

The hybrid MAC protocol, BN-MAC [69], provides a potential solution for mobile nodes and dynamic network patterns. Idle listening time is reduced, and packet collision issues are avoided to reserve energy for extended network lifetime. The partial synchronous scheme plays an important role in time delay mitigation to obtain channel access during the contention period. The BN-MAC also leverages the scheduling mechanism to perform conflict-free communication and diminish the overhearing problem. On top of that, several advanced modellings are invented and implemented to collaborate with the BN-MAC protocol to extend the sleep period and shorten the packet forwarding path as much as possible.

- **AP-MAC**

The asynchronous MAC protocol with low duty-cycle and high energy efficiency in [70] provides a feasible solution for a flexible scheduling mechanism based on estimated traffic conditions. The AP-MAC protocol [70] allows each node to wake up randomly according to a predefined wake-up algorithm to avoid failed transmission caused by packet collision. Furthermore, it enables energy conservation via using a low duty-cycling scheme and enhances the transmission efficiency at the same time. In order to establish a reliable connection between the sender node and the receiver node, it is necessary to ensure nodes to convert between the wake-up period and sleep period as scheduled in advance. AP-MAC leverages the advantage of the adaptive low duty-cycling scheme to make communications both robust and resilient.

- **SLACK-MAC**

SLACK-MAC [71] is proposed using low duty-cycling with maximum 1%. In order to mitigate the possibility of transmission collision or cross-talk effect, the time scheduling of active period and inactive period is designed based on past experiences. The history of successful packet delivery has a crucial effect on the prediction of the subsequent sleep-wake scheduling design. Obviously, the time slot distribution among all nodes is not uniform, unlike random access. However, it is reported that the nodes selected in the past that are successful in data transmission have a relatively higher possibility to work again in the future. After a thorough evaluation process, the improved SLACK-MAC protocol works properly at an extremely low duty-cycle, and the delivery radio can be achieved up to 100% as the pending time spent to generate packets keep increasing. Essentially, the final result of end-to-end delay during effectual transmission process is relatively large (i.e., approx. 300–600 s), which cannot be tolerated in underground CAS design.

#### Scalability

- **SE-MAC**

In SE-MAC [72], the main improvement of communication network scalability is to have time delay mitigated significantly. Therefore, a novel modelling method called Adaptable Application Independent Aggregation (AAIA) was invented to reduce the overall latency. The AAIA model also encompasses cross-layer routing optimisation to further shorten packet delivery delay. The goal of the AAIA model is to make use of constrained power supply and channel bandwidth to perform packet delivery with maximum transmission efficiency using a unique data aggregation scheme. Moreover, there are four different aggregation functions implemented in this model, and they are also capable of alleviating overhead issues.

- **A Hybrid Protocol**

In [73], another solution to extend network scalability was introduced using a hybrid MAC protocol. The communication process provided by this MAC protocol can be divided into two different periods. One is used to contend for channel access and the other one is designed for data transmission. The contention period allows only one device to win the opportunity for pending packets to be sent in the subsequent transmission period using the p-persistent CSMA mechanism. The following transmission period employs the TDMA technique with an improved reservation scheme in channel allocation fairness. The proposed MAC protocol can achieve high performance regardless the size of different wireless sensor networks. Additionally, the refinement of several crucial parameters to balance the contention phase and transmission phase is complicated and has to be adjusted if the target application is changed.

- **SQ-MAC**

The scalable MAC protocol proposed in [74] is focused on the transmission of multimedia data traffic. It enables robust Quality of Service (QoS) support in addition to a limited end-to-end delay in its communication network. SQ-MAC [74] has a random access period to contend for channel access, which is similar with the previous hybrid MAC protocol, and it is subsequent to the scheduled access period using a particular reservation scheme. The reservation-based transmission stage is important to maximise network scalability based on optimum channel utility. This protocol provides a reliable and resilient solution using an adaptive time scheduling method to reserve time slots for following practical packet transmission. The idea of the switching period is designed for broadcasting and makes each sensor node aware of the time slot assignment of the subsequent transmission period. In order to increase channel throughput and network scalability, all free slots can be occupied without energy waste.

#### Handling Mobility

The design of Depth First Search (DFS) is applied in [75] to perform time slot assignment, and making use of the Fault Tolerant Slot (FTS) enables the protocol to be adjusted under different node patterns [75]. Another method handling mobility and offering a novel approach to cope with dynamic active zones around the mobile nodes based on the specified speed threshold [76]. The MS-MAC provides maximised energy efficiency regardless of static or dynamic application scenarios [76].

#### Wake-Up Radio Enabled

In [77], a low-power device TICC1200 (i.e., short for CC1200) was used to keep listening and detect node mobility. It is also capable to wake up ultra-wideband (UWB) devices over a relatively long operation range to perform two-way ranging promptly. Although the system in [77] is designed for inventory management, and devices are mounted on an unmanned aerial vehicle which has higher flexibility in terms of nodes movement compared with V2V or V2P, collisions occurred in underground mines. The employment of external wake-up devices alleviates the crowded resource occupation caused by control frames for the time scheduling mechanism. It also provides mitigated overall time delay and system capacity.

## 5. Summarised Features and Properties

In order to summarise all kinds of features in accordance with their inherent properties, here, we present a set of tables to present the consolidated information involved with the typical CAS design for underground mines.

- *Summary of Commercially Available CAS Products*
- *Summary of Proximity Detection Technologies*
- *Summary of Positioning Techniques*
- *Summary of LPWA Communication Technologies*

PS: The importance of features in each row gradually decreases from top to bottom.

- *Summarised Useful Information Obtained From Existing MAC Protocols*

PS: The importance of features in each column gradually decreases from left to right.

## 6. Discussion

### 6.1. Ranging Process

In order to build a high-performance CAS in the underground mine, we have to determine the choice for proximity detection technology among a wide range of options that are capable of relatively long operating ranges and high measurement accuracy with minimum deployment effort. In essence, the solution must be able to avoid false alarms as much as possible and perform ranging/localisation accurately and rapidly. The false alarm problem may impose a habitual thought on mine workers, and people will easily become used to false alarm signals. When the accident occurs, the worker probably cannot make any prompt reaction to prevent collisions. In addition, the higher frequency of the ranging process to be performed implies the higher possibility of vehicles/personnel in the proximity that can be detected.

Based on previous research work and experimental evaluation, our research group has proved that UWB-based detection technology outperforms other available candidates due to its exceptional cost-effectiveness [78,79]. Impressively, the UWB technique can achieve the accuracy of centimetre level across the measurement range up to 200 m under the Line of Sight (LOS) situation [80]. The duration of the ranging process takes only several nanoseconds, which can be leveraged to provide enough flexibility when considering how often devices should perform proximity detection [81]. The potential of the false alarm problem is also much less than others. Furthermore, the intrinsic property of the UWB technique avoids interfering as well. UWB devices can be easily deployed in harsh environments at low cost. Lastly, Decawave 1000 (DW1000) is a typical device using UWB technology, which possesses all the aforementioned benefits.

To perform proximity detection, DW1000 is adept at two-way ranging using the TOF principle with high accuracy. Although the TDOA algorithm is also compatible with this device, it will not be selected since no time synchronisation would be obviously preferred in order to preserve energy and improve utility.

### 6.2. Communication System

As an external subsystem that provides necessary assistance, the communication system is dedicated to time frame arrangement to make the ranging process be executed in an organised manner coherently. The proposed solution must be able to work across vast areas that imply the signal coverage should be as large as possible without sacrificing the expected transmission efficiency. An unlicensed spectrum with regards to the frequency operation band that avoids huge subscription cost is certainly important. Additionally, long battery life at low production cost is also a desired feature. In fact, the requirement of low latency is the most significant one compared with the others mentioned before, since timing is of paramount importance in CAS design. Low end-to-end time delay provides the potential for prompt reactions to be conducted by workers, and it also enhances channel utility regarding the transmission protocol.

Consequently, low-power and long-range communication technology is selected, and CC1200 is a typical radio frequency transceiver with high performance in signal coverage and battery lifetime at a relatively low price. CC1200 is a narrowband solution and works in the sub-1 GHz ISM band. Actually, it has an extraordinary low latency (i.e., within microsecond level), which is the most important advantage compared with other LPWA techniques.

To operate collaboratively with the selected device, CC1200, a suitable medium access control method is necessary. Similar to the selection of communication devices, low latency is the most significant requirement as well with respect to MAC protocol design. Otherwise, energy efficiency, network scalability, traffic adaptability and channel throughput also need to be considered. Particularly, in the CAS design, the ability of handling mobile nodes cannot be ignored because most vehicles and personnel in the underground mines often move from one place to another. Especially, the method of enabling wake-up radio offers a possible way for optimisation on top of the single-technique-based communication system.

**Figure 8:**
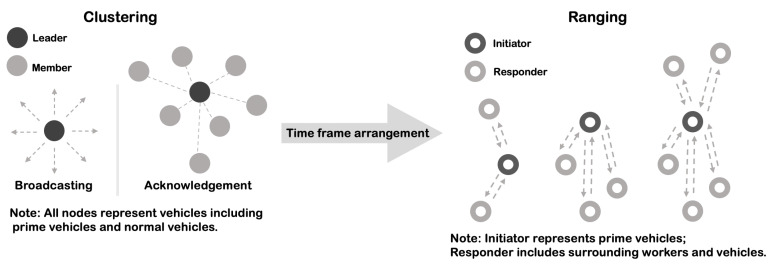
The Workflow of the FTDMA Protocol in the communication system.

From the last summary table in Section 5, the FTDMA protocol is a potential solution for MAC design as proved. It is a conflict-free and cluster-based MAC protocol using the TDMA technique. Since the transmission range covered by CC1200 is longer than that of UWB signals, the communication device would be anticipated to establish stable connections with other devices in the proximity before the detection capability of the UWB device is available. Assume that a unique identity will be assigned to each vehicle or personnel in advance; UWB devices can be easily differentiated with each other throughout the communication. Through the prior message exchange between communication devices next to each other, potential nodes to be involved in the surrounding area will be clustered, and there will be a selected leader based on an appropriate mechanism. The leader is devised to collect and update information for all the member nodes. A flexible time schedule that assigns each unequally divided time slot depending on the real traffic to member nodes would be provided by the leader as well. The leader also broadcasts the time schedule periodically. Afterwards, relevant member nodes send a reply or acknowledgement by order, and the final purpose of the communication system is then achieved. The whole working procedure is thoroughly presented in Figure 8.

Here, in our CAS design, the existence of the communication part is to offer a guidance on time division and arrangement before the task of proximity detection to be performed. However, the nature of UWB signals determines that most LPWA techniques cannot cause interference when they are co-existing with the UWB device in the same area. Thus, there is only one requirement to ensure communication systems work successfully and that is no more than one device has the opportunity to connect with other nodes concurrently. Thereby, the design of the MAC protocol for communication systems is necessary and significant. In general, the communication system is supposed to work independently with the proximity detection system, with the only exception being the time synchronisation problem.

## 7. Conclusions

In this paper, we elaborate an overall review of underground mine CAS design consisting of ranging algorithms and positioning techniques, low-power and long-range communication technologies, as well as appropriate medium access control methods. To this end, a set of summary tables and pertinent discussions are generalised to present the strengths and weaknesses of each option in an organised and concise manner.

Particularly, a comprehensive table that includes most commercially available CAS products in the current market is recapitulated concisely in Table 2. Considering the environment of underground mines, the UWB technique seems to be an effective solution for a range finder, as shown in Table 3. From Table 4, the ranging algorithm of TOF and two-way ranging works decently with UWB devices with the least difficulty in terms of practical implementation. Based on Table 5 and Table 6, a narrowband communication system with an appropriate technology that suits long-range connections at low power, using a TDMA-based scheduling MAC protocol for a distributed wireless network, would be preferred to ensure the determinism of low latency. Therefore, we proposed a potential solution using UWB technology for high ranging accuracy and the TDMA-based MAC protocol for low-latency determinism. In fact, time delay deserves the most significant evaluation metric among multiple feasible solutions. In the other words, short time delay provides the extraordinary superiority of our CAS design when compared with other existing systems. Additional discussion and study on communication protocol design are still ongoing, since the adaptability to resolve various issues that might occur in many complicated application scenarios needs to be focused and enhanced.

## Figures and Tables

**Table 1:** Requirements for the desired MAC protocol design and related works.

Characteristics	Importance	Typical Examples
Distributed network	Mandatory	N/A
Contention-free Communication	Mandatory	N/A
Scheduling Protocol	Optional, good to have	N/A
Latency	Mandatory	SR-MAC, SW-MAC, DW-MAC, LDC-MAC
Energy consumption/overhearing	Mandatory	BBAD Mechanism, RANO Mechanism
Energy consumption/overhead	Mandatory	LO-MAC, LoBigMAC, LCO-MAC
Energy consumption/duty-cycling	Mandatory	BN-MAC, AP-MAC, SLACK-MAC
Scalability	Optional, good to have	SE-MAC, A Hybrid Protocol, SQ-MAC
Traffic Adaptability & Throughput	Optional, good to have traffic adaptability and high throughput	N/A
Handling mobility	Mandatory	N/A
Wake-up radio enabled	Optional, good to have	N/A

**Table 2:** Summary of Commercially Available CAS Products.

Purpose	Company	Product	Technology	Application	Notes	URL
	Mine Site Technologies	Proximity Detection	low-frequency magnetic field	surface & underground mine	detection range: 0.5–20 m	https://mstglobal.com/technologies/safety-tracking/ (accessed on 1 June 2022)
		Situational Awareness	on-board Wi-Fi or Bluetooth tag, RFID tracking tag	surface & underground mine	detection range: 60–120 m	
	NewTrax Technologies	Collision Warning System L7	multiple radio frequency (RF) technologies	underground mine	precise ranging	https://newtrax.com/solution/collision-warning-system-l7 (accessed on 1 June 2022)
		Collision Warning System L8	modular Newtrax Proximity Ranging Sensors (PRS) + L7	underground mine	360 degree awareness & ranging	https://newtrax.com/solution/collision-warning-system-l8 (accessed on 1 June 2022)
		Collsion Avoidance System L9	Same as L8	underground mine	intervention controls	https://newtrax.com/solution-collision-avoidance-system (accessed on 1 June 2022)
	Stanley Black & Decker	AeroScout	active RFID, WiFi (for communication)	underground mine	mobileView software; secure communications based on Cisco unified wireless networks	https://www.cisco.com/c/dam/en_us/solutions/industries/docs/manufacturing/Aeroscout-Cisco-Brochure.pdf (accessed on 1 June 2022)
	Minlog & MapTek	MineSuite	RFID tag (@ 433 MHz), WiFi tag (@ 2.4 GHz)	N/A	RFID: average detection range 50 m; WiFi: detection range, up to 100 m	https://africanminingbrief.com/minlog-deploys-underground-proximity-awareness-bhp-billitons-world-renowned-olympic-dam-mine/ (accessed on 1 June 2022)
CAS	Mine Radio Systems	Helian Underground Safety Solution	UHF RFID Tag, VLF RF communication	underground mine	visual alert via cap-lamp	https://mininglifeonline.net/equipment/mrs-stc-platform/helian/861 (accessed on 1 June 2022)
		underground communication solutions	N/A	underground mine	voice, data and video	https://www.mining-technology.com/contractors/communications/mine-radio/#company-details (accessed on 1 June 2022)
	Industrea Limited	CAS GPS node	GPS, radio transceiver, Bluetooth wireless technology	mining	typically for light vehicles	https://usermanual.wiki/Industrea-Mining-Technology/PROD10522/html (accessed on 1 June 2022)
	Waytronic Security	collision avoidance	camera, ultrasonic detection	manufacturing	Forklift & pedestrian collision avoidance	http://www.wt-safe.com/factorycoll_1.html?device=c&kyw=proximity%20detection%20system&gclid=CjwKCAjwj975BRBUEiwA4whRByB8bQ_ftzM0Zs4B4TWE9d342FB1mn1fTV5bhIOnry_M_8gmXjuehRoCJXcQAvD_BwE (accessed on 1 June 2022)
	InfoTronix	collision avoidance system	VLF magnetic fields	underground mine	special tag arrangement	http://www.infotronix.com.au/productcategory/collision-avoidance-system/ (accessed on 1 June 2022)
	Booyco Electronics	proximity detection, collision warning	RFID: close proximity detection—VLF; long range detection—UHF	underground mine	adjustable warning and danger zones	https://www.booyco-electronics.co.za/product-range/proximity-detection-system-pds (accessed on 1 June 2022)
	Blue Glue (BG)	Third Eye	active RFID Tags	mining	V2P, V2V and V2I (Vehicle to Infrastructure protection)	https://www.blueglue.com.au/products/collision-avoidance-and-proximity-detection/ (accessed on 1 June 2022)
	Blue electronics	Buddy Alert	TOF measuring (@ 2.4 hGHz, 900 MHz), GPS	outdoor	device mounted in vehicles	http://www.blueelectronics.com.au/product_detail/165/PPD-02/ (accessed on 1 June 2022)
	Orbit Communications	Body Guard	i-Tag	outdoor	magnetic mounting	https://www.bodyguardsafety.com.au/proximity-warning-system/ (accessed on 1 June 2022)
	Advanced Mining Technologies (AMT)	CAS-CAM/RF	camera, active sensing—RFID	surface mine	support speed detection	https://www.slideshare.net/nswdre/advanced-mining-technologies-manufacturer-presentation (accessed on 1 June 2022)
	AcuMine	4CAST	GPS and radio frequency signal strength	surface & underground mine	works effectively at both low and high speeds with the same sensitivity	https://im-mining.com/2011/10/19/komatsu-and-acumine-sign-agreement-for-distribution-of-4cast-collision-avoidance-system/#more-4874 (accessed on 1 June 2022)
	PBE	proximity alert system	RFID, GPS, electromagnetics and bidirectional radar	surface & underground mine	combine multiple detection technologies; versatile configurations, suitable for different vehicle types	https://pbegrp.com/safety/proximity-alerts/ (accessed on 1 June 2022)
	Minecom	Dynamic Anti Collision System (DACS600)	UHF RFID tags (operating @ 400 MHz)	mining	None	https://core.ac.uk/download/pdf/39671161.pdf (accessed on 1 June 2022)
	Gamma & Geosteering	TramGuard	low frequency magnetic field	underground, coal mine	fairly short operation range: 3.66 m	https://www.miningmonthly.com/markets/international-coal-news/1303839/massey-demonstrates-proximity-detection-technology (accessed on 1 June 2022)
	EV Alert	collision warning system	VHF short-range coded signal	rail crossing	selected frequency can ‘penetrate’ vehicles and buildings	https://www.parliament.vic.gov.au/images/stories/committees/rsc/Safety_at_Level_Crossing/Submissions/21_EV_Alert.pdf (accessed on 1 June 2022)
CAS	Ivolve	PAMS Proximiti	GPS, radar	mining	long-range, high-speed GPS-based proximity awareness system; short-range, low-speed radar proximity detection capabilities	https://www.mining-technology.com/contractors/resource/ivolve/attachment/ivolve2/ (accessed on 1 June 2022)
	LSM technologies	RadarEye	camera, radar	mining	virtually 360 degree viewing; radar sensor, detection range 2–20 m	https://www.lsm.com.au/item.cfm?category_id=2869&site_id=3 (accessed on 1 June 2022)
	IIT solutions	safe mine system	GPS, radar	mining	a special patented algorithm to calculate the path of vehicles	https://www.australianmining.com.au/product/vehicle-collision-system/ (accessed on 1 June 2022)
		HxGN MineProtect Collision Avoidance System	GNSS, RF technologies	open pit mines	require no support infrastructure	https://hexagonmining.com/solutions/safety-portfolio/collision-avoidance (accessed on 1 June 2022)
	Hexagon Mining	HxGN MineProtect Tracking Radar	tracking radar	open pit mines	operating range up to 30m	https://hexagonmining.com/solutions/safety-portfolio/hxgn-mineprotect-tracking-radar (accessed on 1 June 2022)
		HxGN MineProtect Safety Center	smart camera and combination of above two systems	open pit mines	speed adaptability	https://hexagonmining.com/solutions/safety-portfolio/hxgn-mineprotect-safety-center (accessed on 1 June 2022)
	Minetec	SafeDetect	RF-based mobile nodes, WASP technology developed by CSIRO	surface & underground mine	high-accuracy, low-latency, cm-level proximity detection	http://minetec.com.au/wp-content/uploads/2018/03/MIN-12714-Safedetect-2pp-A4-Flyer.pdf (accessed on 1 June 2022)
	Minewest & Nautilus technology	BUDDY	magnetic field	underground, coal mine	integrated into cap-lamp	http://www.nautilus-intl.com/proximity-detection/nautilus-coal-buddy-operators-proximity-detection-system-for-underground-coal-mines-operating-in-an-explosive-methane-gas-environment-class-i-div-ii/ (accessed on 1 June 2022)
	Becker	collision avoidance system	UHF, radar and electromagnetic field	surface & underground mine	a tri-technology solution	https://www.becker-mining.com/en/products/smartcom/proximity-detection-system-pds (accessed on 1 June 2022)
	Modular Mining	MineAlert	GPS	surface mine	vehicle-to-vehicle only; intelligent path prediction based on vehicle velocity, acceleration and yaw rate	https://www.mining-technology.com/products/minealert-collision/ (accessed on 1 June 2022)
	Matrix Design Group	IntelliZone	magnetic field with optional Lidar/Radar/ camera integration	underground, coal mine	machine-specific straight-line and angled zones	https://www.matrixteam.com/wp-content/uploads/2018/08/IntelliZone-8_18.pdf (accessed on 1 June 2022)
	Preco electronics	PreView	radar, camera	surface & underground mine	various series of products	https://preco.com/product-manuals/ (accessed on 1 June 2022)
	Caterpillar	MineStar Detect	camera, radar, GNSS	surface & underground mine	provide fatigue or distraction detection	https://www.westrac.com.au/en/technology/minestar/minestar-detect (accessed on 1 June 2022)
		Cat Detect	GPS, Bluetooth, WiFi, camera, radar	surface & underground mine		https://www.cat.com/en_US/by-industry/mining/surface-mining/surface-technology/detect.html (accessed on 1 June 2022)
	Strata worldwide	HazardAvert	electromagnetic field	surface & underground mine	programmable at specific speeds	https://www.strataworldwide.com/proximity-detection/surface-and-underground (accessed on 1 June 2022)
		HazardAlarm	electromagnetic field	surface & underground mine	a single-generator system creates a large electromagnetic field	https://www.strataworldwide.com/company/newsroom/alarm-only-proximity-detection-system (accessed on 1 June 2022)
CAS	GE mining	CAS	surface—GPS tracking, RF unit and camera; underground—VLF magnetic and WiFi	surface & underground mine	real-time data connectivity; 12-year proven lifetime	https://www.ge.com/digital/sites/default/files/download_assets/GE-Digital-Mine-Collision-Avoidance-System-datasheet.pdf (accessed on 1 June 2022)
	Jannatec	SmartHelmet	RFID tagging, camera	industrial environments	tailored to each individual customer	https://www.jannatec.com/ensosmarthelmet (accessed on 1 June 2022)
		SmartView	multi-camera, WiFi & Bluetooth (for communication)	mining	voice/text/video communication	https://www.jannatec.com/ensosmartview (accessed on 1 June 2022)
		SmartTalk	N/A	industrial environments	4G LTE radio	https://www.jannatec.com/ensosmarttalk (accessed on 1 June 2022)
	Schauenburg Systems	SCAS surface PDS	RFID, GPS, GSM, camera	surface mine	use time of flight, accuracy <1 m	http://schauenburg.co.za/product/scas-surface-proximity-detection-system/ (accessed on 1 June 2022)
		SCAS underground PDS	cameras	underground mine	tag-less, artificial intelligent	http://schauenburg.co.za/mimacs/ (accessed on 1 June 2022)
		Mine Wide Integrated Monitoring and Control System (MIMACS)	dual-band RF technology	surface & underground mine	2-way Paging & Distress call	http://schauenburg.co.za/wp-content/uploads/2017/03/Schauenburg-MIMACS-Brochure-2017.pdf (accessed on 1 June 2022)
	A&R Engineering	CAS	dual RF technology & time of flight	mining	detection accuracy of better than 1 m to a range of 30 m	http://areng.co.za/collision-avoidance/ (accessed on 1 June 2022)
	Sense technologies	Gaurdian alert	doppler radar	outdoor driving	intended for light vehicles	https://www.businesswire.com/news/home/20050613005521/en/Sense-Technologies-Introduces-Guardian-Alert-ScopeOut-Integrated (accessed on 1 June 2022)
	SICK	proximity sensors	capacitor / magnetic field	manufacturing	N/A	https://www.sick.com/au/en/c/products (accessed on 1 June 2022)
		detection and ranging	Lidar scanning, radar sensing	indoor & outdoor	2D & 3D lidar scanning	
		distance sensors	optic and ultrasonic solutions	positioning	using triangulation and time-of-flight modes	
	Ogden safety systems	Sensor Vision System	multi-beam radar (@ 13.4–14.0 GHz)	quarry vehicles	FMCW principle	http://www.ogdenradar.com/the-radar.php?content=2 (accessed on 1 June 2022)
		VMS (Quarry Vehicle Auto Braking System)				
	Joy Global, P&H (acquired by Komatsu)	Smartzone PDS	electromagnetic field	mining	faceboss integration—easy and quick troubleshooting	https://mining.komatsu/technology/proximity-detection/smartzone-proximity-detection (accessed on 1 June 2022)
		HawkEye camera system	fisheye cameras with infrared filters	mining	Digital Video Recorder (DVR)—100 to 200 h video	https://mining.komatsu/en-au/technology/proximity-detection/hawkeye-camera-system (accessed on 1 June 2022)
	Intec Video Systems	Car Vision	camera	industrial	vehicle safety camera systems	http://www.intecvideo.com/products.html (accessed on 1 June 2022)
		PreView	radar, camera		low power 5.8 GHz radar signal	
	Provix	proximity detection system	RFID, Radar and Sonar object detection	surface & underground mine	N/A	http://provix.net/information/minprodet.asp (accessed on 1 June 2022)
	Septentrio	GNSS receivers	UHF radio, WiFi and Bluetooth (for communication)	mining and construction	N/A	https://www.septentrio.com/en/applications/mining-construction (accessed on 1 June 2022)
CAS	MSHA	MSHA Proximity Detection	electromagnetic field	underground mine	tag-based	http://citeseerx.ist.psu.edu/viewdoc/download?doi=10.1.1.179.1447&rep=rep1&type=pdf (accessed on 1 June 2022)
	Wabtec & GE Transportation	Digital Mine Collision Alert system (CAS)	magnetic field, RF and GPS	surface and underground mine	tag-based	https://www.youtube.com/watch?v=GDFNByOYV60 (accessed on 1 June 2022)
	Ifm Efector	O3M 3D Smart Sensor	optical technology	outdoor	3D image data based on PMD technology	http://eval.ifm-electronic.com/ifmza/web/mobile-3d-app-02-Kollisionsvorhersage.htm (accessed on 1 June 2022)
	Frederick Energy Products	HIT-NOT	magnetic field	warehouse and industry workplaces	N/A	https://hitnot.com/ (accessed on 1 June 2022)
	Rio Tinto (Borax mine)	positioning system	GPS	surface mine	N/A	http://w3.leica-geosystems.com/media/new/product_solution/Dez2004_mining_engineering_GPS.pdf (accessed on 1 June 2022)
	Motion Metrics	ShovelMetrics	radar, thermal imaging	mining and construction	interface with our centralised data analysis platform	https://www.motionmetrics.com/shovel-metrics/ (accessed on 1 June 2022)
	3D Laser Mapping	SiteMonitor	laser scanning	mining	accuracy of 10 mm out of range up to 6000 m	https://www.mining-technology.com/contractors/exploration/3d-laser-mapping/ (accessed on 1 June 2022)
	Hitachi Mining	SkyAngle	camera	mining	bird’s-eye view	https://www.mining.com/web/hitachi-introduces-skyangle-advanced-peripheral-vision-support-system-at-minexpo-international/ (accessed on 1 June 2022)
		Aerial Angle	millimetre wave radar technology	mining	a peripheral vision display system with object detection technology	https://www.mining.com/web/hitachi-construction-machinery-introduces-aerial-angle-peripheral-vision-display-system-with-object-detect-assist-technology-at-minexpo-2/ (accessed on 1 June 2022)
Vision only	Guardvant	ProxGuard CAS	GPS, radar and camera	mining	light vehicles and heavy equipment	https://www.mining-technology.com/contractors/health-and-safety/guardvant/pressreleases/pressguardvant-proxguard-collision-avoidance/ (accessed on 1 June 2022)
	PROXIP	proximity detection system	encoded magnetic field	manufacturing	magnetic field generated by antenna works with electronic marker	https://www.proxipi.com/technologie/?lang=en (accessed on 1 June 2022)
	Safety Vision	vision system	camera	wide range of application	N/A	http://www.safetyvision.com/products (accessed on 1 June 2022)
	ECCO	vision system	camera	wide range of application	N/A	https://www.eccoesg.com/us/en/products/camera-systems (accessed on 1 June 2022)
	Flir Systems	vision system	thermal camera	wide range of application	N/A	https://www.flir.com.au/applications/camera-cores-components/ (accessed on 1 June 2022)
	Nautitech	vision system	thermal camera	harsh environment	marker band identification during cutting cycles	https://nautitech.com.au/wp-content/uploads/2019/05/Nautitech-Camera-Brochure-2019.pdf (accessed on 1 June 2022)
			HD and IR camera	harsh environment	available with Wi-Fi	
		High Bandwidth Networks	N/A	surface and underground mine	fiber optic cables, Wi-Fi APs and mesh	https://mstglobal.com/technologies/network-infrastructure/ (accessed on 1 June 2022)
	Mine Site Technologies	Through-The-Earth Transmission		surface & underground mine	ultra low frequency RF signal	
		Leaky Feeder Radio		surface & underground mine	two-way voice and low-bandwidth data solution	
Communi- cation only	Becker Varis	Vital Alert		underground mine	2-way voice and data; VLF, electromagnetic induction	https://mininglifeonline.net/equipment/our-products/vital-alert/459 (accessed on 1 June 2022)
	Cattron	SIAMnet		underground mine	voice and data; cable modem technology and coaxial cable	http://catce.cl/wp-content/uploads/2019/03/SAIMnet.pdf (accessed on 1 June 2022)
	OTN systems	telecom network for mining		underground mine	N/A	https://www.otnsystems.com/industries/mining (accessed on 1 June 2022)
	MeshDynamics	third-generation of mesh network		surface and underground mine, coal mine	based on the Wi-Fi 802.11 protocol	https://www.meshdynamics.com/documents/Mesh_Mining_July08.pdf (accessed on 1 June 2022)

**Table 3:** Overview and Comparison of Proximity Detection Technologies.

Technology	Operating Range	Distance Accuracy	Update Rate	False Alarms	Interference	Deployment Effort	Operation Condition
Normal Camera	typically >150 m, min 10 m	vision only	real-time	unlikely	unlikely	high	LOS only
Thermal Camera	typically <100 m	vision only	real-time	likely	unlikely	high	LOS only
Infrared Camera	typically <10 m	vision only	real-time	likely	likely	high	LOS only
EM field (approx. 70–140 kHz)	typically 10–100 m (depends on power)	typically m level, ideally <1 m	typically ms level	often	likely	medium	slightly affected by NLOS
Radar	continuous wave: short range; pulsed radar: <30 km	typically submeter level	typically ms level, <70 ms	likely	unlikely	low	LOS only
Lidar	long range: typically >100 m; short range: <50 m	typically cm or mm level	typically ms level, <10 ms	likely	unlikely	low	LOS only
Ultrasonic	typically <10 m	typically submetre level, ideally cm level	typically ms level	likely	unlikely	low	LOS only
RF signals	LF passive (@125 kHz, 134.3 kHz & 225 kHz): typically 10–30 cm, max 2 m; HF passive (@13.56 MHz): typically <1.5 m; UHF passive (@860–960 MHz): 1–50 m; UHF active (@433 MHz): typically 30 m–3 km; SHF active (@2.45 GHz): typically <100 m	proximity only	typically <100 ms (depends on frequency and distance)	often	likely	medium	LF: slightly affected by NLOS; HF: affected by NLOS; UHF & SHF: affected by NLOS profoundly
Bluetooth	Bluetooth 4.0: typically 10–30 m; BLE: average 80 m; long range beacon: typically 200 m	proximity only	Bluetooth: typically 100 ms; BLE: typically 3 ms	likely	likely	low	affected by NLOS
Zigbee	LOS: >300 m; indoor: average <100 m	3–5 m	typically μs level (depends on data rate)	likely	likely	medium	affected by NLOS
UWB	high data rate: <100 m; LOS using IEEE 802.15.4a: <200 m (depends on data rate)	<10 cm (based on ToF)	typically ns level	unlikely	unlikely	low	slightly affected by NLOS

**Table 4:** Overview and Comparison of Positioning Techniques.

Method	Accuracy	Power Consumption	System Capacity	Synchronisation Requirements	Out-Of-Area Positioning
TOF	High	High	Low	No	No
TDOA	High	Low	High	Yes	Yes
TOA	High	Low	High	Yes	Yes
AOA	Low	Low	High	No	Yes

**Table 5:** Overview and Comparison of LPWA Techniques.

Criteria	LECIM	RPMA	LoRa	SigFox	NB-IoT	Telensa	Weightless	LTE-M	IEEE 802.15.4	IEEE 802.11ah
**Latency**	critical message delay: 15 s	typically 10–100 ms	typically 10–100 ms	typically 10–100 ms	typically 10–100 ms	typically 10–100 ms	typically 10–100 ms	typically <150 ms (excluding handover latency)	0–20 s	0–10 s
**Cost**	N/A	Low	Low	Low	High	Low	Low	High	N/A	
**Operation range**	LOS: 20 km; NLOS: 5 km	urban: 15 km; LOS: 500 km	urban: 5 km; rural: 15 km	urban: 10 km; rural: 50 km	urban: 1km; rural: 10 km; typically <15 km	urban: 3 km; rural: 8 km	typically <10 km	typically <11 km	LOS: up to 20 km; NLOS: 5 km	100–1000 m
**Battery lifetime**	typically 3 years	10 years +	10 years +	10 years +	5–10 years	10 years +	10 years +	3–10 years	802.15.4 is more energy efficient than 802.11ah	
**Data rate**	0.00153-125 kbps	uplink: 624 kbps; downlink: 156 kbps	CSS: typically 0.3-5 kbps, up to 10 kbps; FSK: 50 kbps	uplink: 100 bps; downlink: 600 bps	uplink: 64/158.5 kbps; downlink: 128/106 kbps	uplink: 62.5 bps; downlink: 500 bps	downlink: 0.0025-16.0 Mbps; uplink: 0.00025-0.5 Mbps	uplink: 1Mbps, up to 7 Mbps; downlink: 1 Mbps, up to 4 Mbps	0.00153-125 kbps; 250 kbps (in 2.4 GHz)	78 Mbps; 16 Mbps in sub-1 GHz
**Frequency band**	433 MHz	2.4 GHz ISM	sub-1 GHz ISM	sub-1 GHz ISM	7–900 MHz	sub-1 GHz ISM	sub-1 GHz ISM	LTE band	sub-1 GHz ISM; 2.4 GHz (depends on different countries)	sub-1 GHz ISM
**Spectrum license**	unlicensed only in Region 1, not including Australia (Region 3)	unlicensed	unlicensed	unlicensed	licensed	unlicensed	unlicensed	licensed	unlicensed	unlicensed
**Modulation**	DPSK, GFSK	RPMA, DSSS	CSS, FSK	uplink: BPSK, DBPSK; downlink: GFSK	QPSK	2-FSK	QAM, OQPSK, BPSK	uplnik: SC-FDMA, QAM; downlink: OFDMA, QAM	BPSK, FSK, OQPSK	OFDM
**Application**	Critical infrastructure, environmental monitoring	Smart metering, Smart cities, Smart lighting	Smart metering, Smart cities, Smar building	Smart metering, Smart cities, Smart parking	Smart metering	Smart cities, Smart lighting, Smart parking	Smart metering, Asset tracking, Health monitoring	Smart street lighting, environmental conditions monitoring	Smart agriculture, Environment monitoring	Smart Cities, Smart Home

**Table 6:** Summary of Capable Features Based on Existing MAC Protocols.

Year	Protocol	Categorization	Latency	Energy Consumption			Scalability	Traffic Adaptability	Throughput	Handling Mobility	Wake-Up Radio Enabled	Notes
				Overhearing	Overhead	Low Duty Cycle						
1988	E2MaC	contention-free			YES	YES						QoS support
2003	T-MAC	contention-based				YES		YES				balance between overhearing avoidance and maximum throughput
2004	MS-MAC	contention-based			YES	YES				YES		QoS support
2005	S-MAC	contention-based	YES	YES	YES	YES						per-node fairness; collision avoidance
2006	FlexiMAC	contention-free				YES	YES	YES				fair access; data delivery guarantee
2008	DW-MAC	contention-based	YES		YES	YES		YES				aim for bursty and high-traffic loads
	LCO-MAC	contention-based	YES		YES							allow multi-hop transmission within one duty cycle
	eL-MAC	contention-based		YES		YES		YES				suitable for low data rate networks
	SASW-CR	contention-based	YES						YES			UWB-PHY; QoS support
2009	TreeMAC	contention-free			YES				YES			2D frame-slot assignment; for high data rate networks
2010	-	contention-free				YES						aim for low-data-rate WSNs
	VLA-MAC	contention-based	YES			YES		YES	YES			optimised for burst transmission
2011	SQ-MAC	contention-based	YES									multimedia traffic QoS; self recovery
	GLASS	contention-free	YES		YES		YES			YES		aim for data-intensive sensor networks
2012	LDC-MAC	contention-based	YES			YES						dual-channel transmission
2013	-	hybrid					YES		YES			for massive M2M networks
	SR-MAC	contention-based	YES			YES						multi-packet transmission within one operational cycle
	LO-MAC	contention-based	YES	YES	YES	YES		YES				aim for low-data-rate WSNs
	PD-MAC	contention-free							YES			optimisation of scheduling scheme and slot assignment to maximise spatial reuse factor
	FTDMA	contention-free	YES			YES	YES	YES	YES	YES		controlled by cluster heads assigned distributively
	ECOMP	contention-free			YES	YES						clustering; ring configuration
2014	-	hybrid					YES					convention-based CSMA + reservation-based TDMA; QoS support
	SW-MAC	contention-based	YES			YES		YES				scout-based scheduling
	BN-MAC	hybrid		YES		YES				YES		Least Distance Smart Neighbouring Search model
	RANO	contention-based		YES		YES						active RFID protocol
	CT-MAC	contention-based			YES							suitable for direct sequence UWB system
2015	SE-MAC	contention-based	YES		YES		YES					Adaptable Application Independent Aggregation model
	OPC	contention-based	YES						YES			parallel transmission based on local concurrency map
	EH-RDFSA	contention-based				YES		YES				energy harvesting for temporary energy shortages
	H-TSAC	contention-free	YES			YES						hierarchical link scheduling with proactive time slots acquisition
2016	-	contention-free	YES									modifies IEEE 802.15.4; minimum risk of frame collisions
2017	BigMAC	contention-free			YES							receiver-initiated; tree topology
	SLACK-MAC	contention-based				YES						self-adaptive; history-based
2018	AP-MAC	contention-based				YES		YES				self-adaption; collision reconnect mechanism
	BBAD	contention-based		YES				YES			YES	address decoding and validation; increased system capacity

## Nomenclature

2D	Two-dimensional
3D	Three-dimensional
3GPP	Third-Generation Partnership Project
AAIA	Adaptable Application Independent Aggregation
Additive	Increase/Multiplicative Decrease
AOA	Angle of Arrival
AP	Access Point
BBAD	Bit-by-bit Address Decoding
BLE	Bluetooth Low Energy
BPSK	Binary phase shift keying
CAS	Collision avoidance system
CDMA	Code-division Multiple Access
CSMA/CA	Carrier-sense multiple access with collision avoidance
CSMA	Carrier Sense Multiple Access
CSS	Chirp spread spectrum
CTS	Clear to Send
DBPSK	Differential binary phase-shift keying
DFS	Depth First Search
DPSK	Differential phase shift keying
DSSS	Direct-Sequence Spread Spectrum
EM	Electromagnetic
FDMA	Frequency-division Multiple Access
FSK	Frequency shift keying
FTS	Fault Tolerant Slot
GFSK	Gaussian frequency shift keying
GNSS	Global navigation satellite system
GPS	Global Positioning System
GSM	Global System for Mobile Communications
HF	High frequency
IoT	Internet of Things
ISM	Industrial, Scientific and Medical
LF	Low frequency
LoRa	Long range
LOS	Line of Sight
LPWA	Low-Power Wide-Area
LPWAN	Low-Power Wide-Area Network
LTE−MTC	Long-Term Evolution Machine-Type Communication
LTE	Long-Term Evolution
MAC	Medium access control
MIMO	Multiple Input Multiple Output
NB−IoT	Narrow-band Internet of Things
NB	Narrow Band
NLOS	Non-Line of Sight
OFDM	Orthogonal frequency-division multiplexing
OFDMA	Orthogonal frequency division multiple access
OQPSK	Offset quadrature phase shift keying
PION	Pioneer
QAM	Quadrature amplitude modulation
QoS	Quality of Service
QPSK	Quadrature phase shift keying
RANO	Reservation Aloha for No Overhearing
RF	Radio frequency
RFID	Radio-frequency Identification
RPMA	Random Phase Multiple Access
RTS	Request to Send
SCH	Scheduling frame
SHF	Super-high frequency
SRF	Slot-reserved frame
SS	Spread Spectrum
TDMA	Time-Division Multiple Access
TDOA	Time Difference of Arrival
TOA	Time of Arrival
TOF	Time of Flight
UHF	Ultra-high frequency
UMTS	Universal Mobile Telecommunications System
UWB	Ultra-wideband
V2I	Vehicles and infrastructure
V2P	Vehicles and personnel
V2V	Vehicles and vehicles
VLF	Very low frequency
WLAN	Wireless Local Area Network
WPAN	Wireless Personal Area Network
WWAN	Wireless Wide Area Network
